# Inferior vena cava collapsibility index and trauma severity in elderly fall injuries

**DOI:** 10.1590/1806-9282.20240606

**Published:** 2024-09-30

**Authors:** Melike Erden, Huseyin Cahit Halhalli, Asim Enes Ozbek

**Affiliations:** 1 University of Health Sciences, Kocaeli Derince Training and Research Hospital, Department of Emergency Medicine – Kocaeli, Turkey.

**Keywords:** Injury severity score, Falling, Geriatrics, Emergency care, Ultrasonography

## Abstract

**OBJECTIVE:**

Falls are a serious cause of morbidity and mortality among older people. One of the underlying causes of falls is dehydration. Therefore, ultrasonography has become an essential tool for evaluating volume status in the emergency department. However, the effect of volume status on falls in older people has not been evaluated before. The aim of this study was to determine the relationship between the inferior vena cava collapsibility index and the injury severity score in older patients who presented with fall-related injuries to the emergency department.

**METHODS:**

A total of 66 patients were included in the study. The injury severity score was used as the trauma severity score, and the Edmonton Frail Scale was used as the frailty scale. Volume status was evaluated with inferior vena cava collapsibility index. The primary outcome measure was defined as the correlation between inferior vena cava collapsibility index and injury severity score. Secondary outcome measures were defined as the effect of inferior vena cava collapsibility index and injury severity score on hospitalization and mortality.

**RESULTS:**

There was no significant correlation between injury severity score and inferior vena cava collapsibility index (p=0.342). Neither inferior vena cava collapsibility index nor injury severity score was an indicator of the mortality of these patients. However, injury severity score was an indicator of hospitalization. The mean Edmonton Frail Scale score was an indicator of mortality among older people who experienced falls (p=0.002).

**CONCLUSION:**

Inferior vena cava collapsibility index cannot be used to predict trauma severity in older patients who have experienced falls admitted to the emergency department.

## INTRODUCTION

Falls place a significant burden on healthcare systems due to an increase in mortality and morbidity risk among older patients. The underlying reasons for older patients who have experienced falls should be studied in depth to reduce this risk and prevent further fall-related injuries.

A fall is defined as dropping unintentionally or accidentally to a lower level by the effect of gravity^1^. The incidence of falls is 30–40% among community-dwelling older people, whereas it is 50% among those people living in nursing homes. However, this rate is highest among older people with a history of falling in the previous year^2^. The underlying reasons for falls are multifactorial. Several risk factors, such as impaired gait and balance, older age, female gender, the use of assistive devices, arthritis, previous falls, stroke, incontinence, depression, vision problems, orthostatic hypotension, cognitive dysfunction, and polypharmacy, have been reported to be related to falls^3,4,5,6^. Among the older population, drug use is directly proportional to comorbidities. Over-the-counter drug use is common among this population. Antihypertensives, diuretics, and alpha-blockers are related to falls because of volume depletion^7^. Therefore, determining the volume status of older people is important. In emergency department (ED) settings, volume status should be determined with a non-invasive, easy-to-perform, and repeatable method. The inferior vena cava collapsibility index (IVCCI) is a reliable indicator of volume depletion and low central venous pressure^8^.

We hypothesized that the effects of polypharmacy and physiologic changes on volume depletion due to senescence are related to trauma severity. In this prospective study, the relationship between the IVCCI and the injury severity score (ISS) was evaluated.

## METHODS

### Study design

This single-center, prospective, observational study was conducted in an academic emergency department with approximately 250,000 ED visits annually. The study was approved by the local ethics committee (2021/114). Written informed consent was obtained from all participants included in the study.

### Study order and population

The study was conducted with patients over 65 years old who presented to the ED between October 2021 and June 2022 due to falling. Such patients who agreed to participate in the study were included. Non-cooperative and unstable patients were excluded from the study. Patients who required urgent treatment and who were referred to the ED from another hospital were also excluded.

### Study protocol

Written informed consent was obtained from all patients eligible for the study. Demographic data, vital signs, drugs, clinical findings, and risk factors for falling were recorded on predesigned data sheets by an attending physician. The Edmonton Frail Scale (EFS) was used to determine the level of frailty, and the ISS was used to establish trauma severity^9^. The hospital data record system was used to determine whether the participants had been readmitted.

The study investigator was informed when eligible patients came. The study investigator was blinded to the clinical findings of the patients. Fluid resuscitation was not given to the patients before the sonographic evaluation. Sonographic evaluations were performed by an emergency physician with 4 years of experience in basic and advanced ultrasonography. The ultrasonographic examinations were conducted using the Esaote Mylab Gamma^®^ device (Genoa, Italy) and the Esaote AC2541 curved array (1–8 mHz) probe (Genoa, Italy) when the patients were in a supine position. The transducer was positioned on the subxiphoid region in the transverse plane, with the indicator turned to the patient’s right. As the abdominal aorta and inferior vena cava came into view, the transducer was rotated 90° clockwise. Inferior vena cava diameters during expiration and inspiration were measured 2 cm distal from the right atrium in motion mode (M-mode). The IVCCI was measured using the following formula: (IVC expiratory diameter-IVC inspiratory diameter)/IVC expiratory diameter × 100.

### Outcome measures

The primary outcome measure was defined as the correlation between the IVCCI and ISS scores. The secondary outcome measures were defined as the effect of the IVCCI and ISS on admission to the hospital and 30-, 60-, and 180-day mortality rates.

### Statistical analysis

Statistical analyses were performed using SPSS version 25.0 (IBM Corporation, Armonk, NY, USA). The normality of the distribution of data was determined by the Kolmogorov-Smirnov test. The homogeneity of the variances was examined using the Levene test. Continuous variables were presented as the mean and standard deviation (SD) or the median and interquartile range (IQR), and categorical variables were presented as percentages. The continuous variables that showed a normal distribution were analyzed with a Student’s t-test, whereas the data lacking normal distribution were compared using the Mann-Whitney U test. The chi-squared test was used to compare the categorical variables. Fisher’s exact test was used when more than 20% of the cells had anticipated frequencies of less than five. Multivariate logistic regression analyses were used to determine the distinctive factor(s) between the hospitalized and discharged groups and the surviving and exitus groups. All variables for which the p-value was determined to be <0.10 because of univariate statistical analyses were included in the regression models as risk factors. The odds ratio, 95% confidence intervals, and Wald statistics were also calculated for each variable. A p-value of <0.05 was considered statistically significant. The Spearman correlation test was used to determine the relationship between the IVCCI and the ISS.

## RESULTS

A total of 74 older patients were admitted for fall-related injuries during the study. Eight patients who met the exclusion criteria were excluded from the study. Thus, the study was conducted with 66 participants. The participants’ demographics and clinical findings are presented in Table 1.

**Table 1:** Demographic and clinical characteristics of participants.

	All cases (n=66)	Survived (n=54)	Death (n=12)	p-value
Age (median, IQR)	77.6 (65–98)	74 (65–98)	88 (66–94)	**0.002**
Gender (%)				
Female	42 (63.6)	39 (72.2)	3 (25)	**0.006**
Male	24 (36.4)	15 (27.8)	9 (75)	
Comorbidity (%)	62 (93.9)	51 (94.4)	11 (91.7)	0.561
Medication usage (%)	60 (90.9)	50 (92.6)	10 (83.3)	0.298
Fall risk factors (%)				
Previous fall	32 (48.5)	24 (44.4)	8 (66.7)	0.283
Environmental	30 (45.5)	27 (50)	3 (25)	0.210
Tremor-dizziness	23 (34.8)	19 (35.2)	4 (33.3)	>0.999
Hearing problem	17 (25.8)	13 (24.1)	4 (33.3)	0.489
Existing disease	13 (19.7)	9 (16.7)	4 (33.3)	0.232
Pain on movements	5 (7.6)	3 (5.6)	2 (16.7)	0.221
Vision problem	3 (4.5)	1 (1.9)	2 (16.7)	0.083
Injured area (%)				
Extremity-pelvis	35 (53)	28 (51.9)	7 (58.3)	0.931
Head-neck	22 (33.3)	19 (35.2)	3 (25)	0.737
Thorax	8 (12.1)	7 (13)	1 (8.3)	>0.999
Face	7 (10.6)	7 (13)	0 (0)	0.334
Abdomen	6 (9.1)	6 (11.1)	0 (0)	0.582
External structures	0 (0)	0 (0)	0 (0)	

Data are presented as number (%) for the categorical variables. IQR: interqartile range. Bold text indicates significance.

There was no correlation between the IVCCI and ISS scores (p=0.342). Age and male gender were directly related to 30-, 60-, and 180-day mortality (p<0.05) (Table 2). The median ISS was statistically significantly higher in the hospitalized group than in the discharged group (p<0.001). The EFS score was directly related to 30-, 60-, and 180-day mortality (p=0.038, 0.03, and 0.002, respectively). The mean of the IVCCI and EFS values did not differ significantly between the discharged and hospitalized groups (p>0.05). The IVCCI levels did not differ significantly between those who died and survived within 30, 60, and 180 days (p=0.243, 0.775, and 0.901, respectively). ISS did not differ significantly between those who died and survived within 30, 60, and 180 days (p=0.638, 0.681, and 0.646, respectively).

**Table 2:** Comparison of surviving and exitus groups, discharged and hospitalized groups based on injury severity score, inferior vena cava collapsibility index, and Edmonton Frail Scale scores.

	ISS (median, IQR)	IVCCI ≥50 (%)	EFS score (mean±SD)
All cases (n=66)	4 (0–13)	14 (21.2)	6.5±3.2
Discharged (n=41)	2 (0–12)	12 (29.3)	6.2±3.4
Hospitalized (n=25)	9 (0–13)	2 (8)	7.0±2.8
p-value	**<0.001**	0.082	0.349
Survivor (n=54)	4 (0–13)	11 (20.4)	5.9±3.1
Death (n=12)	3 (0–9)	3 (25.0)	9.0±2.4
p-value	0.646	0.708	**0.002**

Data is presented as mean ± standard deviation or median [interquartile range] for the continuous variables and number (%) for the categorical variables. IQR: interquartile range; IVCCI: inferior vena cava caval index; ISS: injury severity score; EFS: Edmonton frailty score. Bold text indicates significance.

Multivariate logistic regression analyses were used to determine the factor(s) distinctive between the hospitalized and discharged groups and the surviving and exitus groups (Table 3). ISS was a distinctive factor that differentiated the hospitalized and discharged groups. The results of the study showed that each one-point increase in ISS increased the probability of hospitalization 1.516 times (95% confidence interval [CI] 1.206–1.907) (p<0.001). Gender and age were the most distinctive factors that differentiated the surviving and exitus groups. When adjusted for other factors, the 180-day mortality risk for males was 12.808 times higher than for females (95%CI 2.183–75.157) (p=0.005). Additionally, regardless of other factors, the 180-day mortality risk increased statistically significantly with age (odds ratio=1.118; 95%CI 1.013–1.234 and p=0.027). The EFS, which was significantly related to 180 days of mortality as a result of univariate analyses, lost its significance when adjusted for age and gender (odds ratio=1.772; 95%CI 0.886–3.544 and p=0.106) (Table 3).

**Table 3:** Examination of the effects of all possible factors thought to be effective on hospitalization and mortality, together with multivariate logistic regression analysis.

	Odds ratio	95%CI	Wald	p-value
Hospitalization				
Hearing problem	3.898	0.811–18.733	2.885	0.089
Extremity	1.652	0.356–7.655	0.411	0.521
ISS	1.516	1.206–1.907	12.672	**<0.001**
IVCCI ≥50	0.398	0.059–2.684	0.896	0.344
Mortality (180 days)				
Age	1.118	1.013–1.234	4.867	**0.027**
Male factor	12.808	2.183–75.157	7.978	**0.005**
EFS score	1.772	0.886–3.544	2.618	0.106

IVCCI: inferior vena cava caval index; CI: confidence interval; ISS: injury severity score; EFS: Edmonton frailty score. Bold text indicates significance.

## DISCUSSION

The results of this single-center, prospective, observational study showed that IVCCI cannot be used to predict the severity of fall-related injuries in older patients. Additionally, neither IVCCI nor ISS was an indicator of the 30-, 60-, and 180-day mortality of these patients. However, ISS was an indicator of hospitalization for fall-related injuries in older patients. Furthermore, owing to the very high risk of mortality attributable to age and gender, physicians should be vigilant for older patients who present to the ED with fall-related injuries.

Although dehydration is very common among older people, there is no gold-standard method for the diagnosis of these patients. The Cochrane Database Group reported that no single method was accurate enough for the determination of dehydration in older patients^10^. However, IVCCI was not evaluated in this study. Orso et al. reported that bedside ultrasonography might have allowed for the rapid and right determination of the hydration status of older patients in the ED^11^. Additionally, Sefidbakht et al. reported that IVCCI values were significantly higher in patients with shock than in the control group^12^. Nagae et al. evaluated whether IVCCI might be an indicator of dehydration in nursing homes and found that there was no relationship between them^13^.

In previous studies that evaluated the ISS scores of older trauma patients, different ISS levels were reported. Rau et al. reported that the mean ISS level was ISS 9.3±4.4, whereas Hefny et al. reported that the median ISS level was 4 (1–16)^14,15^. The results of our study were consistent with Hefny et al.’s study results, in both of which median ISS levels were similar. The discrepancy with Rau et al.’s results might be attributable to the designs of the studies in which the patients presented due to motor vehicle accidents were included. In none of the studies, the relationship between the ISS score and hospitalization or mortality was evaluated.

In previous studies, in which the social frailty prevalence of older people was evaluated, Chang et al. reported that the frailty prevalence was 14.9%, and Fhon et al. reported that 21.2% of the older people in society were moderately and severely fragile^16,17^. In our study, the frailty prevalence was found to be 39.4%. The study populations were different in that our study included patients who visited the ED due to falling, whereas, in the previous studies, the frailty of all societies was evaluated, which might be the underlying reason for the difference in the results of the studies.

In this study, age and male gender were the indicators of mortality for the older patients who presented with fall-related injuries, which were consistent with the previous studies^7,18,19^. However, the underlying reason for the high mortality risk of the male gender could not be understood clearly. In this study, although most of the comorbidities and drug usage rates were similar between the two genders, the rate of coronary artery disease and alpha-blocker usage was significantly higher among the male gender, which might be the underlying reason for increased mortality risk among males.

In a previous study, the 1-year mortality rate of older patients who presented with fall-related injuries was reported at 17.4%, whereas in our study, the 180-day mortality rate was 18.4%^20^. This discrepancy might be attributable to differences in the frailty rates of the patients included in the studies. Additionally, the participants of the previous study were nursing home residents, whereas most of the participants in our study were individuals living at home with their relatives, and the quality of care at home could not be thoroughly evaluated.

### Limitations

This study had several limitations. First, this study was a single-center study. Therefore, the results of the study cannot be generalized. Second, the sample size of the study was lower than that of the previous studies. However, since there were no similar studies on this subject, its contribution to the literature cannot be underestimated. Third, in this study, ultrasonography examinations were carried out by one investigator. Since ultrasonography is an operator-dependent and subjective imaging modality, it may lead to interpreter errors. However, the investigator held basic and advanced ultrasonography certificates and 4 years of experience in ultrasonography in the emergency department. Finally, we did not perform echocardiography during the ultrasonography. Therefore, abnormalities that might affect the results of the study, such as valvular dysfunction, pulmonary hypertension, and heart failure, were not evaluated. However, echocardiography might take a long time and might require advanced skills^20^. Therefore, in this study, we measured IVCCI as a more straightforward and practical method.

## CONCLUSION

IVCCI cannot be used to predict trauma severity in older patients who experienced falls and were admitted to the emergency department. However, since the older population is expected to increase in the upcoming years, evaluating the underlying factors and prevention methods of fall-related injuries is prominent. Therefore, future studies should focus on the evaluation of fall-related injuries among the older population with randomized controlled studies in larger populations.
